# Montelukast as a Successful Treatment for Eosinophilic Cystitis in an Asthmatic Woman Patient

**DOI:** 10.12669/pjms.295.3492

**Published:** 2013

**Authors:** Shicheng Yu, Zhigen Zhang, Gonghui Li

**Affiliations:** 1 Shicheng Yu, Department of Urology, Sir Run Run Shaw Hospital, School of Medicine, Zhejiang University, Hangzhou, P. R. China.; 2 Zhigen Zhang, Department of Urology, Sir Run Run Shaw Hospital, School of Medicine, Zhejiang University, Hangzhou, P. R. China.; 3 Gonghui Li, Department of Urology, Sir Run Run Shaw Hospital, School of Medicine, Zhejiang University, Hangzhou, P. R. China.

**Keywords:** Eosinophilic cystitis, Montelukast, Therapeutics, Asthma

## Abstract

Eosinophilic cystitis (EC) is a rare inflammatory disorder. We herein report a case of EC in an asthmatic female patient who had a recent exacerbation with none known allergen. She was administered montelukast sodium orally for four weeks and received complete remission. This medication was successfully discontinued after a three-month follow up period. This case report about successful treatment of an adult EC patient using montelukast sodium may provide a new option for EC patients with allergic history.

## INTRODUCTION

Eosinophilic cystitis (EC) is a rare inflammatory disorder first reported by Brown in 1960 who described the condition as eosinophilic granuloma of the bladder.^1^ The most common symptom complex consists of frequency, urgency, dysuria, hematuria and suprapubic pain. Although the cause of EC remains unclear, etiological factors including parasitic infection and intravesical instillation of chemotherapeutic agents (Mitomycin or Candthiotepa) or BCG have been reported.^2^^-^^4^ Eosinophilic tissue infiltration is a heterogeneous group of diseases involving the respiratory system, gastrointestinal tract and skin. It is hypothesized that EC could be one kind of the eosinophilic tissue infiltration diseases, and eosinophilic infiltration in related organs like the respiratory tract could have comorbid disease in the urinary tract.^5^ To support this hypothesis, we herein report a case of EC in an asthmatic female patient who had a recent exacerbation with none known allergen.

Most EC cases reported previously were treated with corticosteroids or antihistamines, but the effective rates were variable.^6^ Sterrett et al^7^ reported their experience in the treatment of EC with leukocyte antagonist (montelukast sodium) in a 6-year-old child, whose symptom remission remained dependent on this medication. In our case, montelukast sodium was administered for four weeks and discontinued successfully after a three-month follow up period. This case report about successful treatment of an adult EC patient prompts montelukast sodium as an alternative for the current treatment modalities of EC patients with an allergic history.

## CASE REPORT

**Fig.1:**
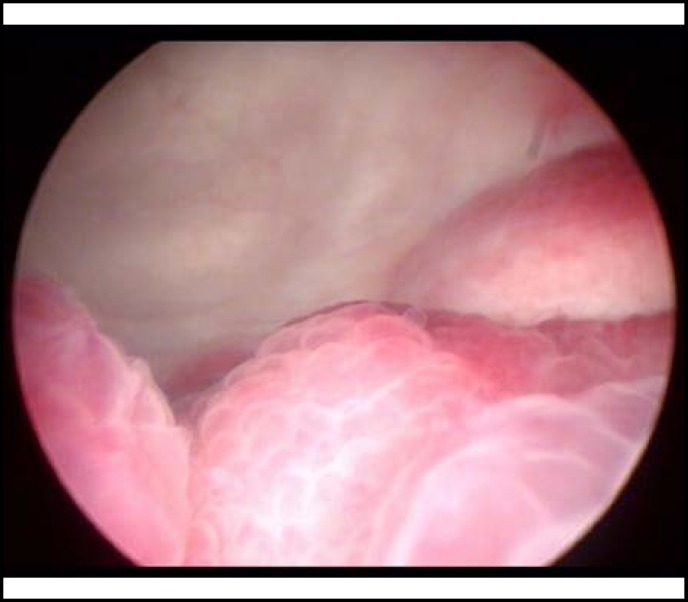
A velvety and polypoid lesion involving the right ureteral orifice

**Fig.2:**
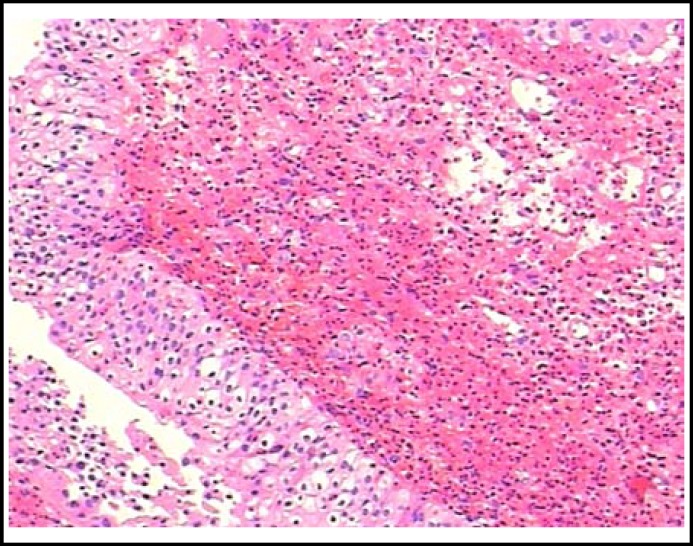
Transmural inflammation predominantly with eosinophilic infiltration into the lamina propria (HE stain, ×40 magnification).

A 47-year-old woman was admitted to our hospital presenting with gross hematuria, dysuria and suprapubic pain during micturition, and she had a moderate asthma exacerbation at the same time. Her daytime urinary frequency ranged from 15 to 30 times with nocturia awaken about 4 to 6 times. Physical examination was not remarkable except for suprapubic tenderness. Laboratory tests showed eosinophilia (2.64×10^9^/L, 30.9%) with normal white blood cell (WBC) count (6.77×10^9^/L). Urinalysis showed RBC 5-10/HPF while the chemical profile and repeat urine cultures were normal. Ultrasound scan revealed a 4.4cm×4.8cm×1.6cm irregular mass continuous with the bladder wall with mild dilation of the right renal collecting system. Cystoscopy revealed an erythematous, velvety and polypoid lesion involving the right ureteral orifice with hyperemic mucosa in the trigone [Fig.1]. Biopsy specimens taken from the lesion showed transmural inflammation predominantly with eosinophilic infiltration, associated with focal muscle necrosis and fibrosis [Fig.2]. 

Seeing that the patient had asthma exacerbation at the same time, she was administered with montelukast sodium (10mg/d) and flavoxate orally. Her urinary symptoms were relieved gradually, and asthma was well controlled. After four-week treatment, a repeat cystoscopy showed that the bladder mass had disappeared. Montelukast sodium was discontinued after a three-month follow-up period when the urinary symptoms were relieved without evidence of recurrence.

## DISCUSSION

Eosinophilic cystitis is a rare inflammatory condition characterized by transmural eosinophilic infiltration of the bladder wall. EC affects adult men and women equally, although a slight male preponderance was reported among children.^6^

Patients of EC usually present with irritative voiding symptoms and sterile urinary culture. Physical examination is usually unremarkable. Radiological findings include bladder occupying lesion that mimics invasive bladder carcinoma, and dilation of the upper urinary tract are usually found in cases with ureteral orifice involvement. Peripheral eosinophilia is found in nearly 40% of EC patients, which could aid in the diagnosis. Although the clinical manifestation and radiological imaging are non-specific, a confirmed diagnosis can be achieved via cystoscopic biopsy.^6^^,^^8^

As most EC biopsy specimens show transmural eosinophilic infiltration and peripheral eosinophilia has been reported in several cases, allergy is hypothesized to be a putative initiator for EC. It is postulated that the offending antigen enters the bladder to stimulate B lymphocytes to produce IgE which binds to tissue mast cells. Mast cells coated by IgE are sensitized and later exposed to the same antigen, resulting in degranulation and secretion of leukotriene (LTC4 and LTD4) and histamine, which act on the surrounding tissues. The antigen-antibody complex also stimulates Th2 lymphocytes to release interleukin 5 (IL-5) and eotaxin, which recruit eosinophils. The eosinophils will secret cationic protein when activated by leukotriene and IL-5. The eosinophil cationic protein enhances the inflammatory reaction and damages the detrusor muscle, leading to fibrosis. As local allergy of the bladder due to direct contact with the allergen is rare (only mitomycin C or thiotepa reported), it is presumed that EC may be part of the tissue eosinophilic infiltration disease. Asthma is the allergic airway inflammation which can also cause systemic eosinophilia. The cytokine-rich environment in asthmatic patient may stimulate the chemotaxis of eosinophils in the urinary tract. In our case, the patient was diagnosed with EC when she was experiencing an asthma exacerbation, which may support the above hypothesis.

In view of its self-limiting tendency but a high risk of recurrence, management of EC is usually conservative. Symptom control and fast reduction of inflammation are advocated.^8^ Corticosteroids have been successfully utilized in several EC cases due to their strong anti-inflammatory effect by decreasing eosinophilic action and preventing phospholipid release.^9^ But the adverse effects of corticosteroids including withdraw syndrome, osteoporosis, and peptic ulcer limit their clinical use in EC treatment. Leukotrienes formed by inflammatory cells are pro-inflammatory mediators, playing an important role in eosinophil recruitment and activation in EC. Leukotriene receptors have been found in inflammatory cells, the respiratory tract, skin and human detrusor muscle. Montelukastis a leukotriene receptor antagonist and has been used for the treatment of asthma. It may also be effective for EC treatment due to its selective inhibition on leukotriene receptor mediated response.^10^ Sterrett et al^7^ reported successful control of the disease by using montelukast in an EC child, although symptom relief remained dependent on this medicine. In our case, the patient was treated with montelukast and finally withdrew the medication without recurrence, which may serve as a clue to support its role in EC treatment.

Eosinophilic cystitis is likely to be misdiagnosed as other urinary disorders such as invasive bladder cancer due to its unspecific clinical features, though the diagnosis can be confirmed by cystoscopic biopsy. Medical treatment to reduce inflammation as soon as possible is recommended. Montelukast appears to offer protection to the urothelial tissue and an anti-inflammatory response by inhibiting eosinophil activation, especially in EC patients with an allergic history of asthma.
